# Recombination and the role of pseudo-overdominance in polyploid evolution

**DOI:** 10.1101/2025.02.28.640841

**Published:** 2025-03-06

**Authors:** William W. Booker, Daniel R. Schrider

**Affiliations:** 1 Department of Genetics, University of North Carolina at Chapel Hill, Chapel Hill, North Carolina, 27514-2916, United States of America

**Keywords:** Polyploid, overdominance, pseudo-overdominance, associative overdominance, inbreeding, population genetics

## Abstract

Natural selection is an imperfect force that can under some conditions fail to prevent the buildup of deleterious mutations. Small population sizes and the lack of recombination are two such scenarios that reduce the efficiency of selection. Under these conditions, the disconnect between deleterious genetic load and individual fitness due to the masking of recessive deleterious mutations in heterozygous individuals may result in the emergence of pseudo-overdominance, wherein the buildup of haplotypes with complementary sets of deleterious mutations results in apparent heterozygote advantage and an increase in linked neutral diversity. In polyploids, the presence of additional allelic copies magnifies this masking effect and may therefore increase the probability of pseudo-overdominance. Here, we simulate the evolution of small diploid and autotetraploid populations to identify the conditions that support the evolution of pseudo-overdominance. We discover that pseudo-overdominance evolves under a much wider range of parameters in autotetraploids than in diploids with identical population sizes, and that in many parts of parameter space there is an inverse relationship between fitness and recombination rate. These results imply that pseudo-overdominance may be more common than previously thought. We conclude by discussing the current evidence for pseudo-overdominance in species with polyploid histories, as well as its implications in agriculture due to the prevalence of polyploidy in crops.

## INTRODUCTION

Recombination is a fundamental process in shaping the evolution of species and their genomes. In its absence, mutations accumulate on chromosomes without the ability to be purged outside of the destruction of the entire chromosome’s lineage (Muller 1964; Felsenstein 1974). When present, recombination breaks down linkage disequilibrium and new favorable mutations can find their way onto common alleles while deleterious mutations can be removed from those backgrounds, enabling a more efficient traversal to the peaks of the fitness landscape (Crow and Kimura 1965; Hill and Robertson 1966; Felsenstein 1974; Barton 1995). One might therefore expect selection to drive recombination rates higher and higher. However, plants and animals are generally limited to having roughly two crossovers per generation per chromosome—albeit with substantial variation across species (Wilfert et al. 2007; Stapley et al. 2017; Brazier and Glémin 2022). This phenomenon is in part due to molecular constraints such as crossover interference (Muller 1916; Wang et al. 2015; Ritz et al. 2017), but may also result from the deleterious effects of too much recombination: recombination events can break up haplotypes that have accumulated multiple beneficial alleles (termed ‘recombination load’, Charlesworth and Charlesworth 1975; Barton and Charlesworth 1998).

Pseudo-overdominance presents an extreme evolutionary phenomenon in which evolving higher rates of recombination is maladaptive (Frydenberg 1963; Sved 1968, 1971, 1972; Ohta and Kimura 1970; Ohta 1971, 1973; Charlesworth 1991; Becher et al. 2020; Waller 2021). Pseudo-overdominance occurs when extremely low recombination rates result in the buildup of deleterious mutations on disparate haplotypic backgrounds causing heterozygotes with alternate high-load haplotypes to be the most fit. Because mutations are unlikely to occur independently at the same site, low recombination regions of the genome can accumulate high levels of mutational load when mutations are at least partially recessive and therefore the load is only expressed in the homozygous state. This will result in an excess of genetic diversity relative to the neutral expectation (Pamilo and Pálsson 1998), not only for the deleterious mutations themselves but also for linked neutral alleles (with the latter effect said to be a consequence of associative overdominance; Frydenberg 1963). When pseudo-overdominant dynamics are operating, selection will disfavor mutations that increase recombination rates (Pálsson 2002), suggesting that selection could also favor modifiers that decrease recombination under pseudo-overdominance.

Pseudo- and associative overdominance have received relatively little attention, but signatures of these dynamics have been detected in *Drosophila* (Latter 1998; Becher et al. 2020) and humans (Gilbert et al. 2020), and have been suggested as a mechanism shaping the heterogeneous density of genetic diversity and load across the genomes of additional plant and animal species (Waller 2021). Although theory suggests these dynamics are most likely to occur in smaller, inbred populations (Zhao and Charlesworth 2016), they may also occur in larger populations under some conditions. For example, population bottlenecks can push populations with larger census sizes into a pseudo-overdominant state (Bierne et al. 2000). Moreover, even in large populations that have not experienced bottlenecks the reduced *N*_*e*_ (effective population size) of low recombination regions caused by background selection and selective interference with other loci under selection could also subject these regions to the conditions in which pseudo-overdominance dynamics can occur (Zhao and Charlesworth 2016; Charlesworth and Jensen 2021).

The equilibrium state of a finite diploid population under pseudo-overdominant dynamics is that of multiple haplotypes with complementary deleterious mutations segregating in the population at frequencies relative to s (the selection coefficient) after alternative haplotypes have been lost to drift (Gilbert et al. 2020). Effectively, this means that although individual fitness is high, roughly half of all offspring are homozygous for a high-load haplotype. However, although rarely considered, diploidy is not the sole ploidal state in biology, and tetraploids under the same equilibrium dynamics would produce between 0–25% homozygote high-load offspring depending on the allelic state of the parents (with the maximum fraction of high-load offspring produced by mating between two AAAa parents, where A and a represent the two haplotypes). Higher-level ploidies would produce even fewer high-load offspring.

Polyploidization has shaped the evolutionary history of nearly all branches of the tree of life (Otto and Whitton 2000; Gregory and Mable 2005; Breuert et al. 2006; Wood et al. 2009; Albertin and Marullo 2012; Zhan et al. 2016; Barker et al. 2016; Van de Peer et al. 2017; Li et al. 2018; One Thousand Plant Transcriptomes Initiative 2019). Although there is considerable debate over any paradigmatic evolutionary trajectory of polyploids, several generally acknowledged maxims of polyploid formation may promote pseudo-overdominant dynamics in polyploids. First, due to the nature of polyploidization, all newly formed polyploids go through an initial population bottleneck significantly lowering their effective population size (Stebbins 1950). Second, the multiplication of chromosomes in a cell causes widespread genomic instability presenting several challenges to undergoing regular meiosis, and evidence suggests there exists a strong pressure to evolve lower recombination rates in newly formed polyploids (Morrison and Rajhathy 1960; Reddi 1970; Wu et al. 2014; Bomblies et al. 2016; Morgan et al. 2021)–often below that of their diploid progenitors (Mulligan 1967; Yant et al. 2013). Finally, polyploids are more strongly associated with self-fertilization and asexual reproduction than diploid taxa (Barringer 2007; Stenberg and Saura 2013; Neiman et al. 2014), processes which increase inbreeding load and therefore the potential for pseudo-overdominance as discussed above. Combined with the relative rarity of homozygotes in polyploids compared to diploids at identical allele frequencies and the corresponding greater masking of deleterious alleles, these observations suggest pseudo-overdominance may play an elevated role in polyploids as compared to other taxa.

In this study, we investigate the conditions in which pseudo-overdominant dynamics are present in autotetraploids. Using forward-in-time simulations, we analyze the relationship between fitness and demography as a function of ploidy, population size, mutation rate, recombination rate, and the strength of selection. We conduct simulations under a wide variety of parameters, attempting to identify the parameter space where pseudo-overdominant dynamics are favored in diploid and autotetraploid populations. Additionally, we describe the complex relationship between fitness and recombination rate in small populations, as well as the long-term trajectory of those populations.

## METHODS

### Simulations model overview

All simulations were conducted in SLiM version 4.0.1 (Haller and Messer 2017, 2023) with modifications to simulate tetraploids as done in Booker and Schrider (2024), where the full details of our simulation model can be found. Briefly, because SLiM does not support polyploids natively, we created polyploids by initializing two populations where one population functions as a storage population for the extra two chromosomes in each tetraploid, and the other population stores the other two chromosomes and is used to track the demographic and selection processes throughout the simulation. All polyploids were modeled as tetrasomic autotetraploids, with chromosomes only forming bivalents during meiosis (i.e. chromosomes pair up for recombination and segregation during meiosis, rather than in groups of 3 or 4). When forming bivalents, all possible pairings of the 4 chromosomes were equally likely. For these simulations there was no burn-in period as this period is generally intended to get populations to equilibrium conditions, and the purpose of this study is to describe the nature of reaching equilibrium and if that equilibrium condition is pseudo-overdominance.

### Demographic and fitness model and associated parameters

Simulations were conducted under a constant-size Wright-Fisher (WF) model. Individuals in the simulation have a single chromosome 1Mb in length, and the per-base pair mutation and recombination rates are constant across all sites. For all simulations, population size N is constant and an individual’s fitness relative to that of all individuals in the population determines the probability of that individual being chosen for reproduction. Fitness is calculated multiplicatively according to Eq. 1:

Eq. 1 $$w=\prod_{i}^{n}\;\left(1\;*\;s_{i}\right)$$

Where w is an individual’s fitness, and s is the selection coefficient for a given homozygous mutation i. For all simulations mutations were fully recessive and s was constant across all mutations in a given simulation.

We varied all parameters of the simulation and analyzed multiple combinations of population size (N), selection coefficient (s), recombination rate (⍴), and the mutation rate (μ) to understand the interplay of these parameters in driving pseudo-overdominance in diploid and tetraploid populations. Distributions of these parameter values were chosen based on initial tests to capture the range of values where pseudo-overdominance could occur given the other fixed parameters. Due to the large number of parameter combinations, simulations were limited to 10 replicates per unique set of parameters.

## RESULTS

### Fitness is stable and high in autotetraploids under pseudo-overdominant dynamics

**Figure 1.**
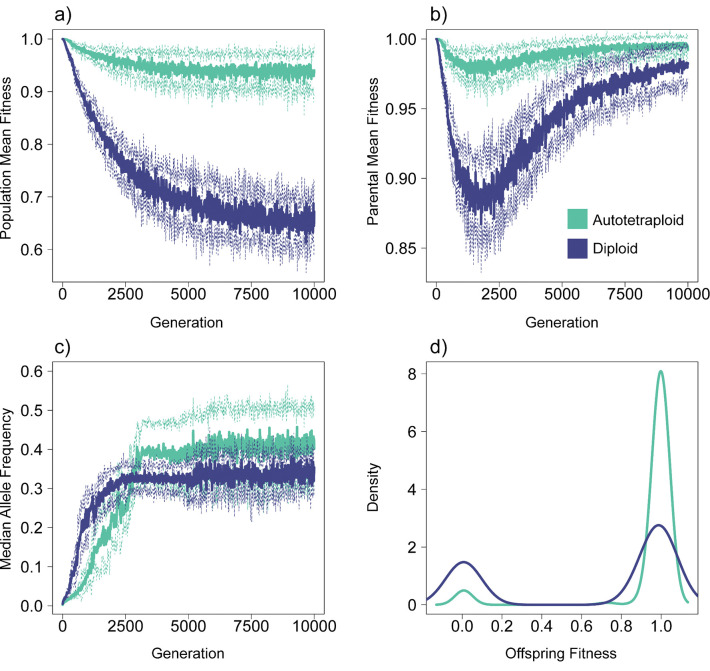
Differences between diploids and autotetraploids under pseudo-overdominant dynamics. a) Mean fitness of the total population at each generation. Solid lines represent the average across 10 simulations, with dashed lines representing ±1 standard deviation. Note that these values are not relative fitness, but SLiM’s absolute fitness calculation based on all segregating deleterious mutations present in an individual. b) Mean fitness of parents chosen for reproduction at each generation. c) Median allele frequency at each generation. d) Density of offspring fitness for diploids and tetraploids at generation 10,000 of the simulation. The parameters for the simulation replicates shown in this figure are *N* = 100, *μ* = 1.0e-07, *s* = −0.005, ρ = 1.0e-10.

We first characterize and describe the differences between autotetraploids and diploids under pseudo-overdominant dynamics. Using an illustrative example (*N* = 100, *μ* = 1.0e-07, *s* = −0.005, ρ = 1.0e-10), the fitness of both autotetraploids and diploids decreases prior to reaching equilibrium pseudo-overdominant dynamics (Fig. 1a); however, population fitness decreases much more in diploids and continues to decrease well after pseudo-overdominant dynamics are occurring (~ generation 2000, inflection point in parental mean fitness curve, Fig. 1b). In contrast to the overall population fitness, Fig. 1b shows that the fitness of individuals sampled for reproduction recovers after average allele frequencies reach equilibrium dynamics (~ generation 2500 here, Fig. 1c) in both diploids and autotetraploids, despite the fact that the fitness difference between heterozygous and homozygous individuals continues to widen after this point (as seen in the different fitness trajectories for the population as a whole and those selected to be parents in Fig. 1a,b). The cause of the difference in fitness between ploidies under pseudo-overdominance is evident from the distributions of offspring relative fitness values in autotetraploids and diploids (Fig. 1d). While eventually the accumulation of mutations results in fitness approaching zero in homozygotes of both diploids and tetraploids, the distribution of offspring genotypes in the two groups is drastically different, with a much smaller proportion of low-fitness homozygotes in tetraploids. The higher average allele frequency in tetraploids may partially account for this, but the smaller probability of forming homozygotes in tetraploids plays a greater role. For example, with an allele frequency of 0.4 (which is the approximate median at equilibrium for tetraploids in Fig. 1c) we would expect 52% of offspring genomes to be homozygotes in a diploid population vs. ~16% in a tetraploid population.

### Pseudo-overdominant dynamics occur over a wider range of parameters in autotetraploids than in diploids

We observed that simulations exhibiting pseudo-overdominant dynamics such as the bimodal offspring fitness distributions shown in Fig. 1d always had intermediate median allele frequencies at equilibrium (e.g. ~30%–~40% in the examples shown in Fig. 1), while simulation replicates not experiencing pseudo-overdominance had much lower median allele frequency (typically ~0–10%). We therefore use the presence of intermediate median allele frequency at equilibrium as a proxy for pseudo-overdominance in our description of our simulation results below.

**Figure 2.**
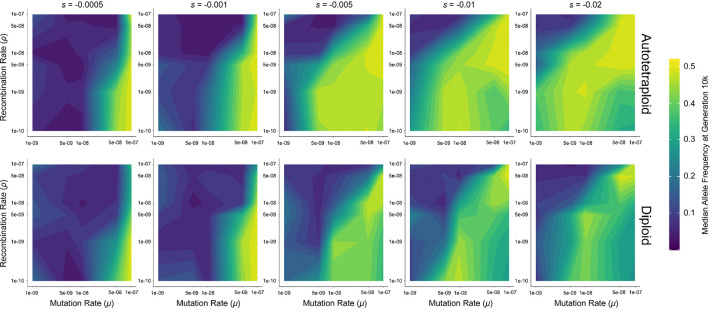
Median allele frequency at 10,000 generations for autotetraploid (top) and diploid (bottom) simulations of a population of *N* = 100 individuals. Axes are transformed to log scale, with values between ticks interpolated to fill in the gradient. Allele frequencies for each parameter combination are averaged across 10 replicates.

For both diploids and autotetraploids, pseudo-overdominance tends to occur as the recombination rate decreases and while the mutation rate and selection coefficient increase, although we find evidence of pseudo-overdominance in high recombination rate models under many parameter combinations (Fig. 2; Suppl. Fig. 1–2). However, the relative area of parameter space where intermediate median allele frequencies are observed is larger in autotetraploids and diploids, indicating a wider range of conditions under which pseudo-overdominance is expected in autotetraploids. Although it appears at *N* = 500 that at low selection coefficients diploids occupy a greater pseudo-overdominant parameter space (*s* = −0.005 and *s* = −0.001 at generation 10,000, Supp. Fig. 2), looking at the median allele frequency over the course of the simulation shows that at a higher *N*, it takes longer for autotetraploids to enter pseudo-overdominance, and autotetraploid populations have not hit equilibrium by generation 10,000 (Supp. Fig. 7–8). Nonetheless, at larger selection coefficients (*s* = −0.1 or −0.2), pseudo-overdominant dynamics are already more prevalent for autotetraploids at generation 10,000 when *N* = 500, and autotetraploids more readily exhibit pseudo-overdominance at higher recombination rates than diploids (e.g. *μ*=5e-8 and ρ=1e-7, Supp. Fig 7–8).

**Figure 3.**
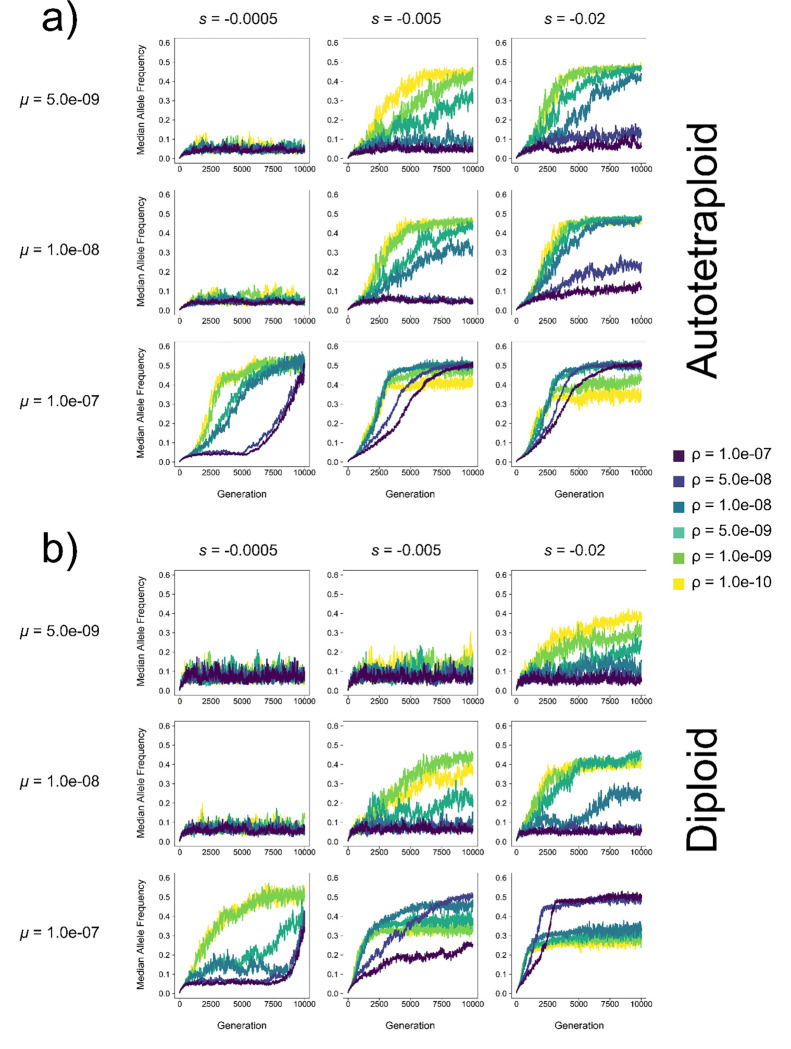
Median allele frequency of (a) autotetraploids and (b) diploids in each generation at varying mutation rates (*μ*) and selection coefficients (s) for a population of *N* = 100 individuals. Each line represents the average across 10 replicates.

Although for larger populations autotetraploids transition to pseudo-overdominance more slowly than diploids, the speed of this transition can vary substantially across parameter combinations. For example, autotetraploids typically reach pseudo-overdominance before diploids in small populations (i.e. *N* = 100; Fig. 3). The sharpness of the transition also varies in a manner that likely depends on the magnitude of fitness differences between offspring as a small number of haplotypes with alternative mutations begin to dominate the population. In some cases there is a gradual approach to equilibrium, such as the simulations with ρ=1e-8, *s*=−0.005, and *μ*=1e-8 in Fig 3a. For others, there appears to be little initial change in allele frequencies followed by a sudden rapid increase towards equilibrium, such as the high recombination simulations at *s* = −0.0005, with *μ =* 1.0e-07 (Fig. 3a,b). These sudden transitions appear to be more common in conditions where pseudo-overdominance is especially likely: high mutation rates and strong selection with low recombination rates and small population sizes.

A necessary consideration, however, is that snapshots at individual generations where median allele frequencies are high are not necessarily indicative of a population in pseudo-overdominance. Median allele frequencies fluctuate rapidly when *μ*, s, and *N*, are low, and do so to a greater extent in diploid populations which have half the number of chromosomes in the population (Fig. 3b, Supp. Fig. 2–8). This dynamic is particularly exaggerated at *N* = 100, where the allele frequencies in low *μ* populations fluctuate from ~0 to nearly 0.5 in diploids. However, because these frequencies are not stable, pseudo-overdominance is not the source of their elevated state. Because of this, the estimates of pseudo-overdominant parameter space from Fig. 2 and Supp. Figs. 1–2 likely overestimate the area of pseudo-overdominance in diploids relative to autotetraploids. The difference in proclivity to over-dominance between autotetraploids and diploids may thus be even greater than our results suggest, at least in smaller populations.

**Figure 4.**
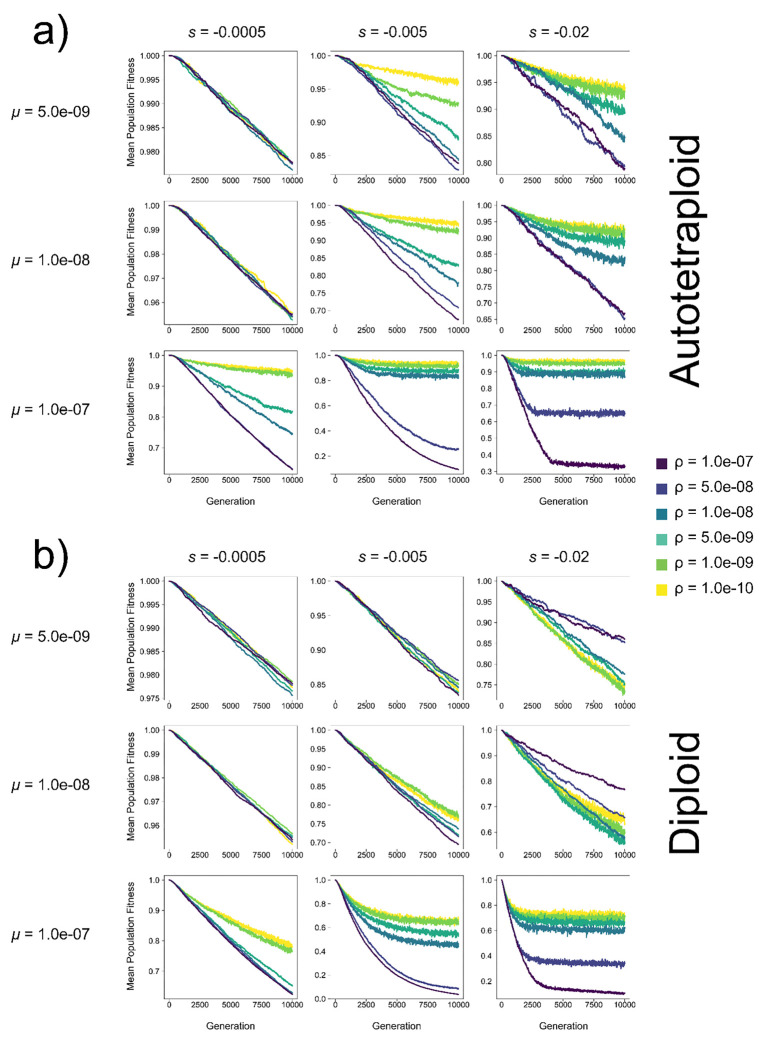
Mean population fitness of (a) autotetraploids and (b) diploids in each generation at varying mutation rates (*μ*) and selection coefficients (s) in a population of *N* = 100 individuals. Each line represents the average across 10 replicates. Note: y-axis varies across subfigures.

Finally, a striking result demonstrating the fitness benefits underlying pseudo-overdominance is that for several combinations of *μ*, s, and *N* the recombination rate is inversely proportional to fitness in both diploids and autotetraploids, and the range of parameter space where this fitness-recombination relationship occurs is much greater in autotetraploids (Fig. 4, Supp. Figs. 9–14). Furthermore, for autopolyploids in any specific combination of *N*, s, and *μ*, where pseudo-overdominance does occur at some ρ, a reduction in ρ is either selectively neutral or advantageous in nearly all cases. The same is not true for diploids, where there is often a fitness cost in reducing ρ from 1.0e-7 (1 recombination per chromosome per generation) to an intermediate ρ before the benefits of pseudo-overdominance are realized in lower recombination rates (e.g. high s simulations in Supp. Figs. 10,12,14); while similar trends are seen in some instances for autotetraploids, the differences in fitness across recombination rates are much smaller in magnitude in such cases (e.g. *μ*=5e-9 and *s*=−0.02 in Supp. Figs. 13, 15). In total, this result illuminates the routes to pseudo-overdominance in both diploids and autotetraploids but demonstrates that the path is clearer in autotetraploids.

## Discussion

### Pseudo-overdominance is expected to be far more prevalent in polyploids than diploids

As evolutionary processes, pseudo- and associative overdominance are generally considered to be rare—at least in part because the conditions necessary for entering those dynamics in diploids require specific combinations of several population genetic parameters that makes for a narrow part of parameter space in which these conditions can be satisfied (Charlesworth and Jensen 2021). Gilbert et al. (2020) showed that under a multi-locus model, this parameter space is wider than previously thought. Here we show that for organisms of higher ploidy the range of conditions where pseudo-overdominance is expected is dramatically expanded. As such, pseudo-overdominance may be a much more prominent evolutionary force than currently appreciated.

The main cause for the increased relevance of pseudo-overdominance in polyploids is rather straightforward: the masking effect of additional allelic copies allows polyploid organisms to tolerate a greater load of recessive deleterious mutations—effectively lowering the barrier of entry into pseudo-overdominant parameter space. As demonstrated here, fitness remains high in polyploids both prior to and after entering into pseudo-overdominant dynamics (Fig. 1). Conversely, in diploids the transition to pseudo-overdominance is preceded by a dramatic drop in fitness, and after the transition even those individuals selected to reproduce exhibit much lower fitness than observed in pseudo-overdominant polyploid populations.

### The interplay between pseudo-overdominance and recombination rate

Results from our simulations demonstrate that for both diploids and polyploids, there are combinations of *μ*, s, and *N* where population fitness appears to be inversely proportional to the recombination rate (Fig. 4, Supp. Figs. 9–14). Furthermore, this was observed in across a much wider range of the parameter space in polyploids, with the majority of *μ*, s, and *N* combinations that elicited pseudo-overdominance also demonstrating this inverse relationship. This is consistent with theoretical results demonstrating that pseudo-overdominance occurs when the relative absence of recombination prevents high-load alleles from exchanging deleterious mutations across haplotypes resulting in higher fitness heterozygotes despite that greater load (Frydenberg 1963; Ohta and Kimura 1970; Ohta 1971; Charlesworth 1991).

Previous work on pseudo-overdominance has largely focused on the genomic conditions that support the buildup of deleterious mutations and linked neutral diversity to enter a pseudo-overdominant state, and evidence of pseudo-overdominance has been found or been suggested to occur in some organisms with genomic regions reflecting these conditions (Becher et al. 2020; Gilbert et al. 2020). However, this relationship may be bidirectional, as one might expect pseudo-overdominance to impact the forces that shape recombination rates as well. Indeed, it has been shown that mutations that increase the local recombination rate would be disfavored under pseudo-overdominance (Pálsson 2002), potentially implying that recombination suppressors could be favored. While we did not directly investigate whether recombination modifying alleles would successfully invade under pseudo-overdominant conditions, our results do suggest a clear path through which lower recombination could evolve due to pseudo-overdominance without having to pass through any intermediate recombination rate fitness valley. Moreover, our results suggest that any relationship between pseudo-overdominance and selection on recombination modifiers would be particularly pronounced in polyploids, and that this relationship may contribute to the consistently observed recombination suppression observed in newly formed polyploids (Morgan et al. 2021; Morrison and Rajhathy 1960; Reddi 1970; Wu et al. 2014; Bomblies et al. 2016).

### Wide variation in the timing and abruptness of the transition to pseudo-overdominance

Pseudo-overdominance can only emerge when multiple complementary haplotypes each with a combined large deleterious fitness effect can form while evading selective purging of their constituent deleterious mutations. In many of the simulated scenarios we examined, the transition to pseudo-overdominance occurs relatively rapidly once the simulation begins, suggesting that pseudo-overdominance may often arise immediately following the strong bottleneck of polyploid formation in genomic regions favoring these dynamics (e.g. see most of the low-recombination rate results in Fig. 3a). However, the transition can also occur gradually when the recombination rate is intermediate and intermediate combinations of s and *μ* (i.e. intermediate in both, or high s and low *μ*, or vice versa; diagonals moving from bottom-left to top-right in Fig. 3a, Supp. Figs. 3,5). In such cases blocks of mutations may be sufficiently deleterious to be immediately subject to negative selection, but the recombination rate is not high enough for selection to remove these mutations as fast as they accumulate—an effect compounded by the masking effect of polyploidy. On the other hand, the transition to pseudo-overdominance can be delayed but sudden if blocks of deleterious mutations initially accumulate while largely hidden from selection until there is a tipping point and recombination becomes disadvantageous. For example, such a deferred but precipitous transition occurs if the mutation rate and recombination rates are high, but the selection coefficient and effective population size are small (e.g. the higher-recombination simulations with *s*=−0.0005, *μ*=1e-7 in Fig 3a). We speculate that the onset of pseudo-overdominance may be delayed because in this scenario because individual deleterious mutations are initially purged through the interplay of recombination and selection, but the high rate of mutation eventually results in complementary dense clusters of deleterious mutations on multiple haplotypes. Once formed, the cumulative fitness effects of these dense clusters combined with the low probability of within-cluster recombination would lead to an abrupt transition to strong pseudo-overdominance.

### Evidence of pseudo-overdominance in polyploid species and crops

As discussed above, there are numerous conditions specific to polyploid formation that favor entering pseudo-overdominance: reductions in rho (Morrison and Rajhathy 1960; Bomblies et al. 2016; Morgan et al. 2021), and low census or effective population sizes (Stebbins 1950), perhaps through self-fertilization or periodic asexuality (Pálsson 2001; Barringer 2007; Stenberg and Saura 2013; Neiman et al. 2014). To date, pseudo-overdominance or associative overdominance has not been directly implicated in polyploids. However, there is some circumstantial evidence in salmonids that warrant further investigation: while investigating allele surfing, Rougemont et al. (2023) found that residual non-diploidized autotetraploid regions of the Coho salmon (*Oncorhynchus kitsuch*) genome harbored a far greater deleterious load than diploidized regions, and that this tetraploid-specific load was concentrated in genomic segments with low recombination rates. Furthermore, Bierne et al. (2000) suggested the correlation between heterozygosity and developmental stability in other *Oncorhynchus* trout (Leary et al. 1984) was evidence of associative overdominance in these species. Given these trout experienced the same tetraploidization event as Coho salmon and likely had a similar delayed diploidization, an investigation into the relationship between inheritance, genetic load, and the potential role of pseudo-overdominance in salmonids may be fruitful.

Although *Oncorhyncus* species have considerable economic value as game and food species, the potential role of pseudo-overdominance likely has more widespread agronomic implications, as numerous important crop species are polyploid (Renny-Byfield and Wendel 2014). For many such species, polyploidy itself was critical to the domestication process leading to dramatic phenotypic changes (Salman-Minkov et al. 2016; Zhang et al. 2019b). However, the masking effect of polyploidy on recessive alleles also makes selection difficult as deleterious recessives are hard to remove from the population. Pseudo-overdominance may then compound this problem as any genomic regions under these dynamics will have an even higher genetic load, and high-load individuals can be highly fit but produce offspring with very low fitness. Furthermore, selection in crop breeding typically involves processes that work to reduce *N*_*e*_ or promote heterosis and therefore breeders may inadvertently push more genomic regions into pseudo-overdominant dynamics thereby increasing the genetic load (for a discussion on heterosis and pseudo-overdominance, see Waller 2021).

Again, while pseudo-overdominance has not been directly attributed to genomic signatures in polyploids presently, circumstantial evidence in autopolyploid crops suggest its presence. Perhaps most clearly, genomic signatures and complications with selective breeding in potatoes are consistent with expectations from pseudo-overdominance. Compared with other crops, potatoes have had minimal gains in yield (Douches et al. 1996)—a phenomenon that may be attributable to difficulties in developing homozygous inbred lines in potato because of its high deleterious load (Lindhout et al. 2011). Although the production of self-compatible diploids has resulted in improvements, the residual load from the autotetraploids continues to plague breeders (Wu et al. 2023). Indeed, one inbred potato line retained highly heterozygous regions that accounted for 20% of the genome despite 9 generations of selfing (van Lieshout et al. 2020), and much less dramatic deviations from expected loss in heterozygosity from selfing in maize (which is diploid) were attributed to pervasive associative overdominance (Roessler et al. 2019). Zhang et al.’s (Zhang et al. 2019a) investigation into the origin of inbreeding load in potatoes suggest most of the deleterious load is heterozygous and found in low recombination pericentromeric regions of the genome. Interestingly, much like our simulations showed pseudo-overdominance can occur in high recombination regions when mutations are sufficiently deleterious, Zhang et al. (2019) also found that the most strongly deleterious mutations (including lethals) occurred in high recombination regions and were found in a heterozygous state. Finally, Wu et al. (2023) found that tetraploid potato lines harbored more heterozygous load than diploids, and their work suggest breeders should make selections based on maximizing homozygous load to prevent propagation of the more pervasive but masked heterozygous load.

Though the evidence is less clear than in potatoes, sugarcane is another crop where pseudo-overdominance may be responsible for difficulties in genetic gain. Modern sugarcane is a hybrid of octoploid *Saccharum spontaneum* and decaploid *Saccharum officinarum*, resulting in cultivars that vary in ploidy with between 8 and 12 chromosomal copies and recurrent aneuploidy (Amalraj and Balasundaram 2006). This initial hybridization of multiple species could have promoted pseudo-overdominance in addition to the elevated ploidy (Waller 2021), and although the present work focused on autotetraploids, the contribution of masking towards pseudo-overdominance due to additional chromosomal copies should be more pronounced in higher-level polyploids. Given the complex genome of sugarcane, uncovering strong population genetic evidence of pseudo- and associative overdominance would be challenging, but there are some indications of pseudo-overdominance in this highly polyploid species: Raboin et al. (2008) found pervasive high linkage disequilibrium across the sugarcane genome, and Ferreira et al. (2005) argued against selfing in sugarcane due to its high genetic load resulting in yield losses. Furthermore, the *S. spontaneum* genome has persisted despite repeated backcrossing with *S. officinarum* including the unreduced transmission of 2n *S. officinarum* chromosomes (Healey et al. 2024). Because yield gains have plateaued in sugarcane (Yadav et al. 2020), investigations into the role of pseudo-overdominance may provide insights that can help mitigate these deficiencies.

## Conclusions

Pseudo-overdominance occurs when selection is inefficient in purging recessive deleterious mutations due to small effective population sizes and genomic conditions that increase the disconnect between realized fitness and deleterious load. An increase in chromosomal copy number through whole genome duplication is one process which increases this disconnect. Although pseudo-overdominance is thought to be rare, multi-locus models have shown these dynamics can occur over a wider parameter space than previously appreciated, and our results show that this space is greatly expanded in polyploids. While we have highlighted a few examples of species with polyploid evolutionary histories that have demonstrated evidence of pseudo-overdominance, there are surely many more such cases in present-day polyploids, and recently diploidized genomes could also harbor signatures of past pseudo-overdominance. For example, divergence between duplicates may be elevated in regions previously evolving under pseudo-overdominant dynamics. Alternatively, pseudo-overdominance could drive the process of diploidization itself, resulting in older divergence times as the accumulation of complementary deleterious load favors the suppression of recombination and the divergence of haplotypes into distinct subgenomes. We have focused on crop species here due to the available evidence, but the implications for conservation—where small populations and hidden deleterious load are key concerns—also warrant consideration (Kyriazis et al. 2021). Similarly, although we have focused on autotetraploids as shorthand for polyploids with tetrasomic inheritance, our work is relevant for any polyploid with deviations from strict disomic inheritance—a common occurrence across both auto- and allopolyploids (Li et al. 2021). Ultimately, our work provides a basis for understanding pseudo-overdominant dynamics in polyploids, and more generally implies that pseudo-overdominance may be a more consequential evolutionary force than previously thought.

## Supplementary Material

Supplement 1

## Data Availability

All simulation scripts can be found in the github repository https://github.com/wbooker/polyploid_pod.
