# Supplementary material for: Recombination and the role of pseudo-overdominance in polyploid evolution
Source: bioRxiv. 2025 Mar 6:2025.02.28.640841. Preprint. [Version 1] doi: 10.1101/2025.02.28.640841 (PMC11908147; doi:10.1101/2025.02.28.640841)
Supplement: Supplement 1 [file NIHPP2025.02.28.640841v1-supplement-1.pdf]

bioRxiv preprint doi: https://doi.org/10.1101/2025.02.28.640841; this version posted March 6, 2025. The copyright holder for this preprint (which was not certified by peer review) is the author/funder, who has granted bioRxiv a license to display the preprint in perpetuity. It is made available under aCC-BY 4.0 International license.

SUPPLEMENTAL INFORMATION

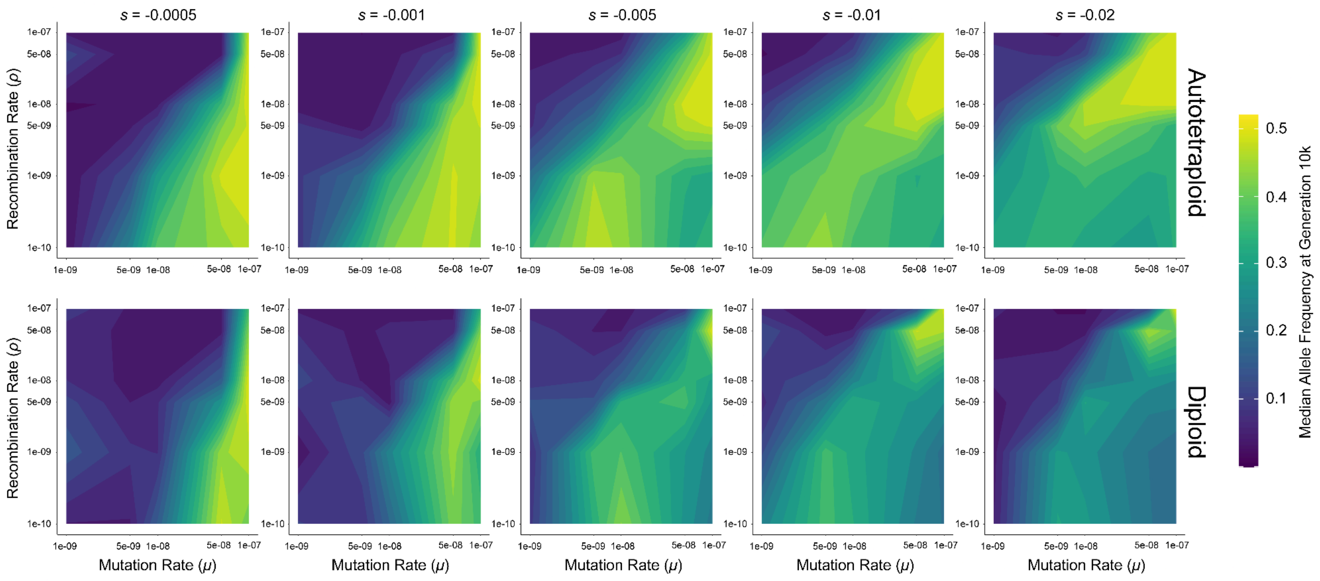

Supplemental Figure 1. Median allele frequency at 10,000 generations for autotetraploid (top) and diploid (bottom) simulations of a population of N = 200 individuals. Axes are transformed to log scale, with values between ticks interpolated to fill in the gradient. Allele frequencies for each parameter combination are averaged across 10 replicates.

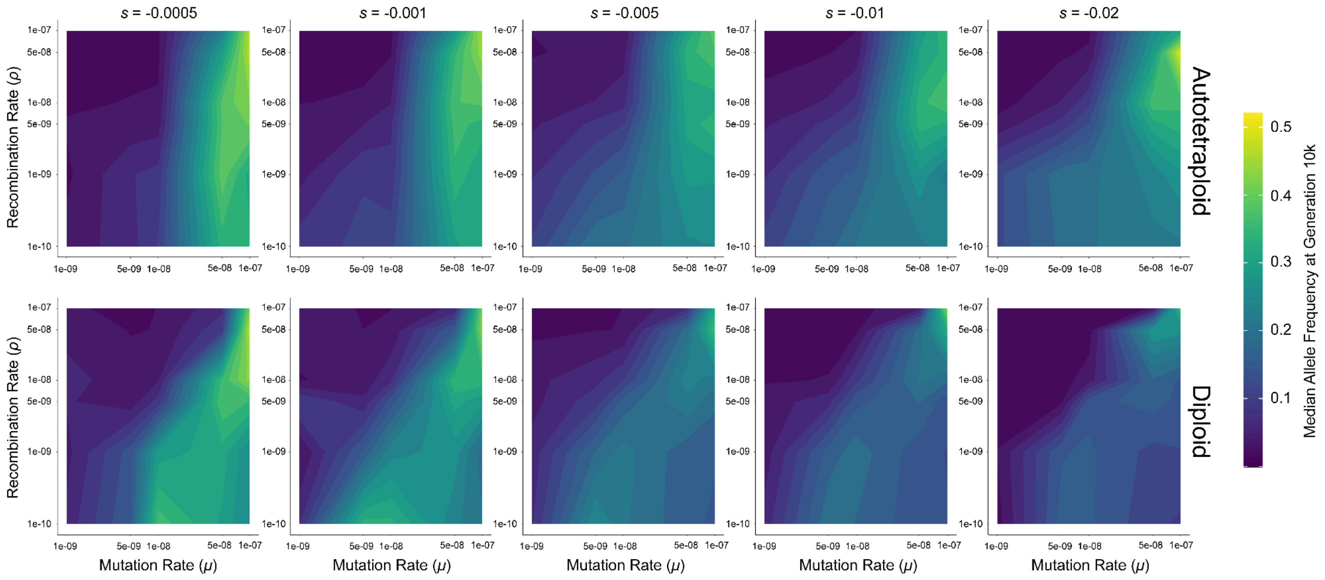

bioRxiv preprint doi: https://doi.org/10.1101/2025.02.28.640841; this version posted March 6, 2025. The copyright holder for this preprint (which was not certified by peer review) is the author/funder, who has granted bioRxiv a license to display the preprint in perpetuity. It is made available under aCC-BY 4.0 International license.

Supplemental Figure 2. Median allele frequency at 10,000 generations for autotetraploid (top) and diploid (bottom) simulations of a population of N = 200 individuals. Axes are transformed to log scale, with values between ticks interpolated to fill in the gradient. Allele frequencies for each parameter combination are averaged across 10 replicates.

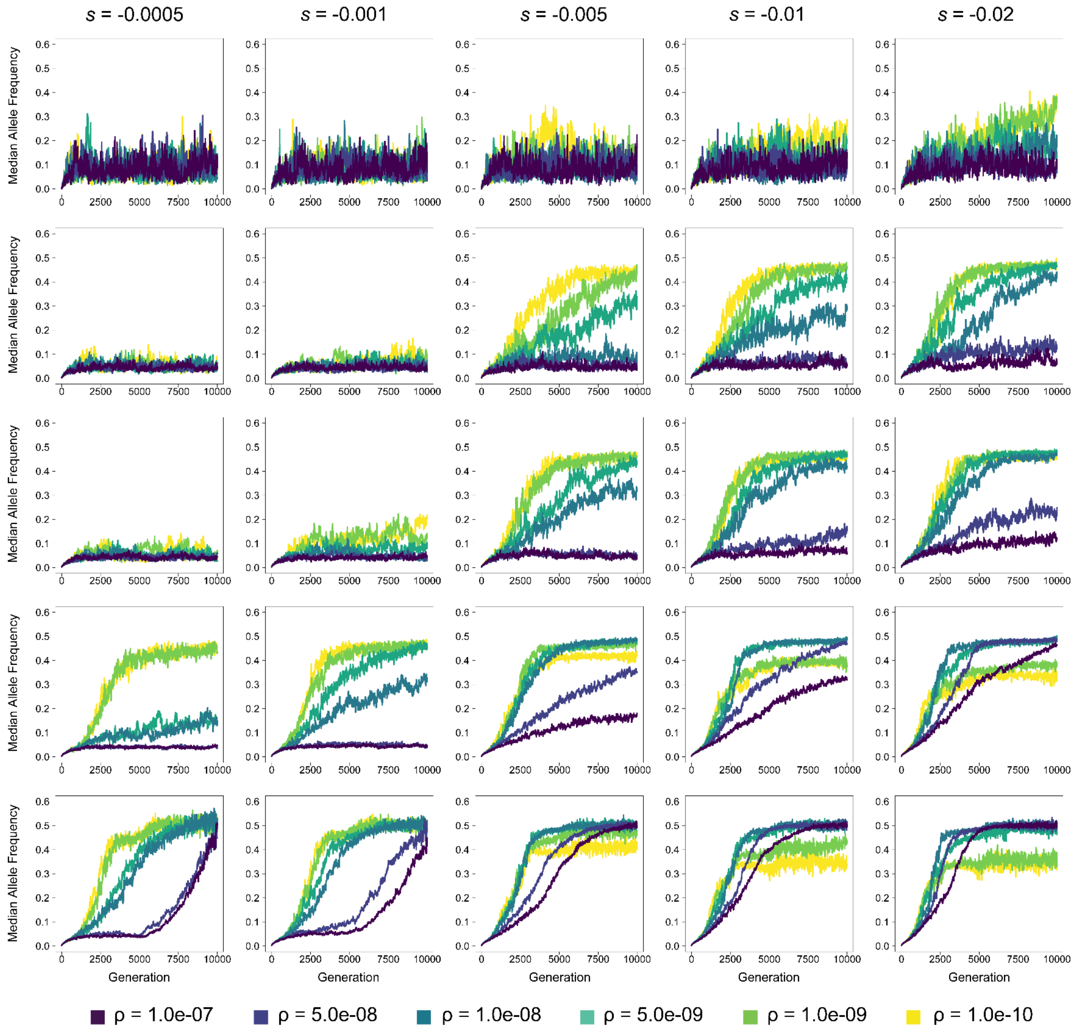

Supplemental Figure 3. Median allele frequency of autotetraploids in each generation at varying mutation rates (μ) and selection coefficients (s) for N = 100. Each line represents the average across 10 replicates. Note: y-axis is identical across subfigures

bioRxiv preprint doi: https://doi.org/10.1101/2025.02.28.640841; this version posted March 6, 2025. The copyright holder for this preprint (which was not certified by peer review) is the author/funder, who has granted bioRxiv a license to display the preprint in perpetuity. It is made available under aCC-BY 4.0 International license.

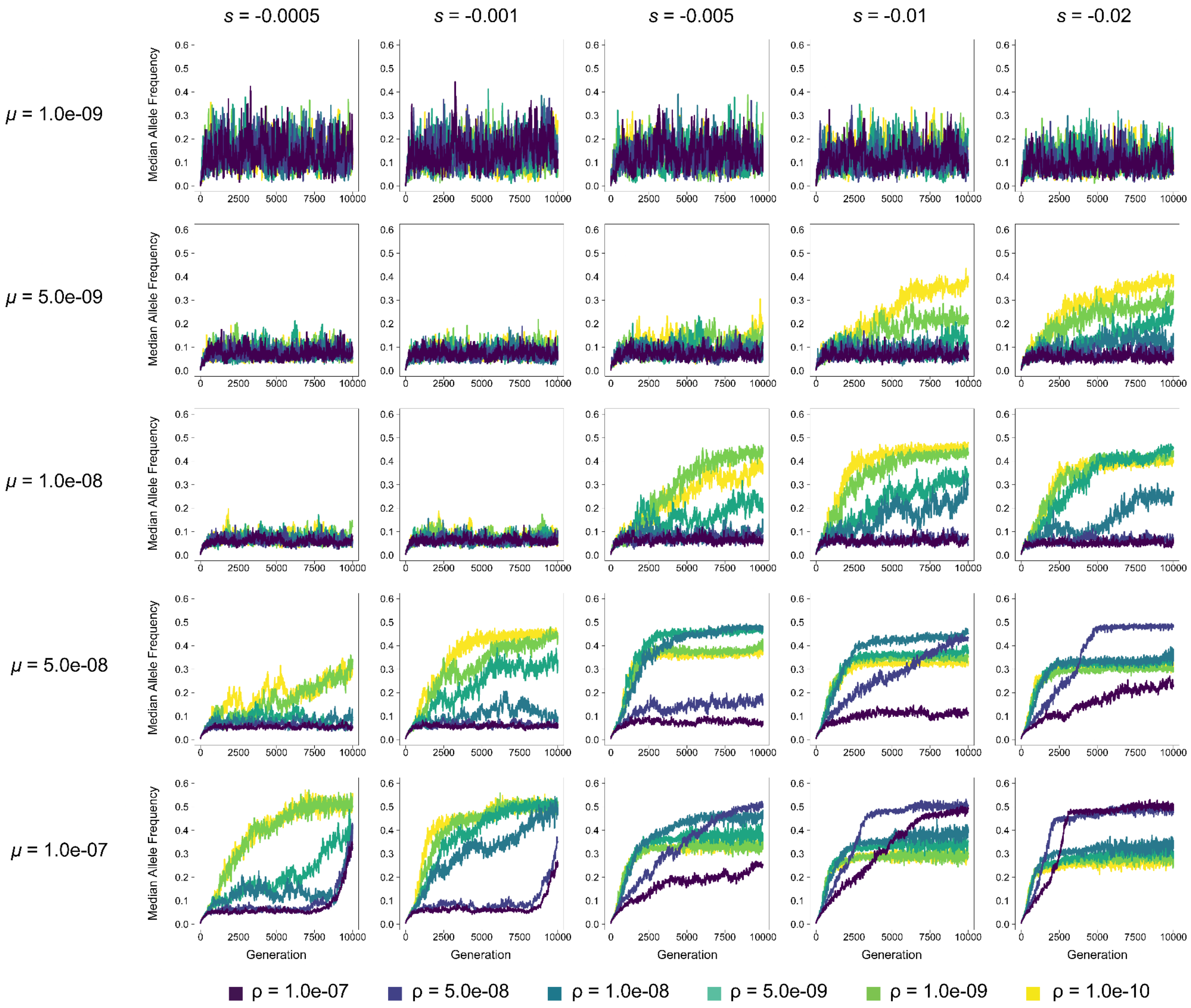

Supplemental Figure 4. Median allele frequency of diploids in each generation at varying mutation rates (μ) and selection coefficients (s) for N = 100. Each line represents the average across 10 replicates. Note: y-axis is identical across subfigures

bioRxiv preprint doi: https://doi.org/10.1101/2025.02.28.640841; this version posted March 6, 2025. The copyright holder for this preprint (which was not certified by peer review) is the author/funder, who has granted bioRxiv a license to display the preprint in perpetuity. It is made available under aCC-BY 4.0 International license.

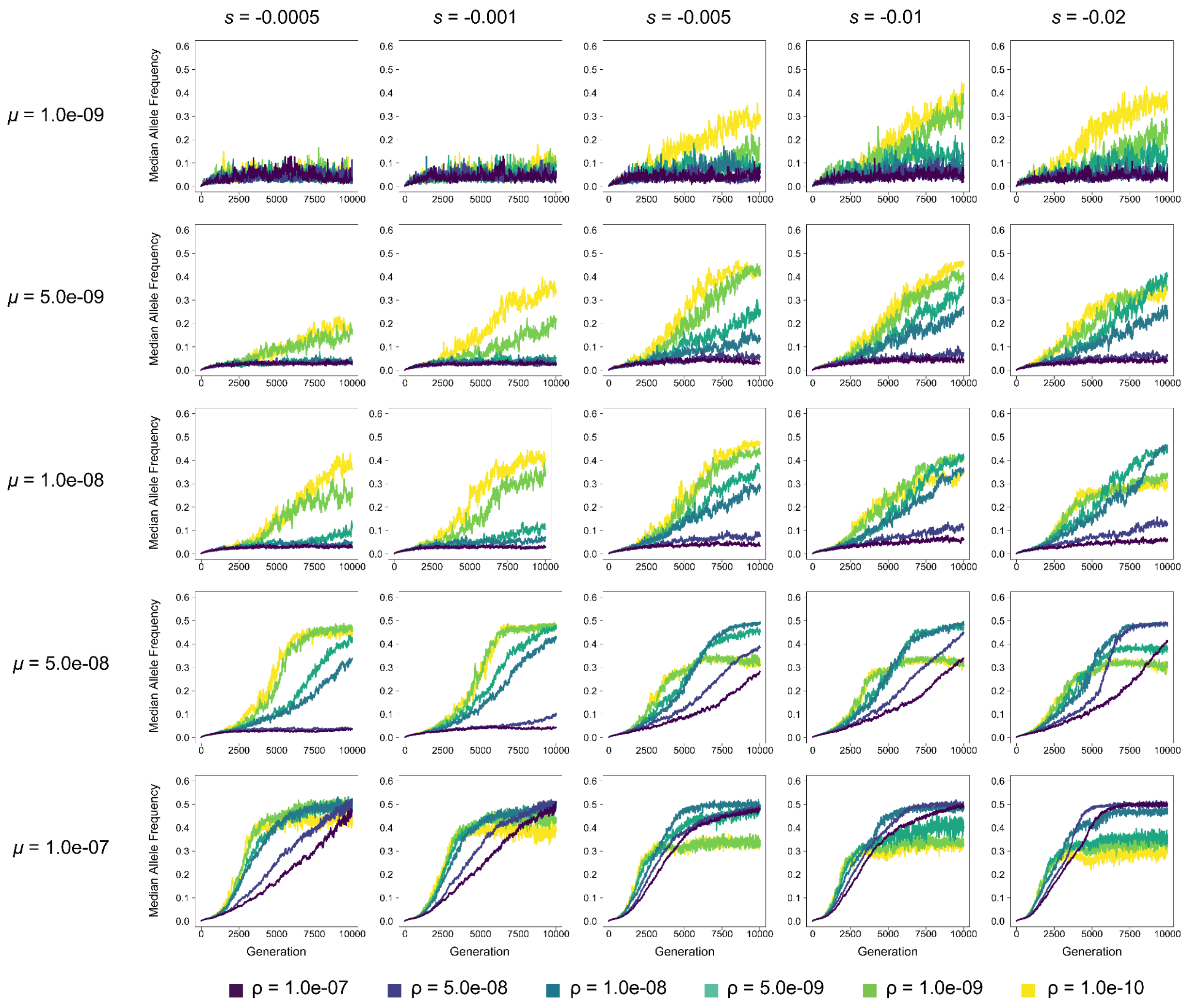

Supplemental Figure 5. Median allele frequency of autotetraploids in each generation at varying mutation rates (μ) and selection coefficients (s) for N = 200. Each line represents the average across 10 replicates. Note: y-axis is identical across subfigures

bioRxiv preprint doi: https://doi.org/10.1101/2025.02.28.640841; this version posted March 6, 2025. The copyright holder for this preprint (which was not certified by peer review) is the author/funder, who has granted bioRxiv a license to display the preprint in perpetuity. It is made available under aCC-BY 4.0 International license.

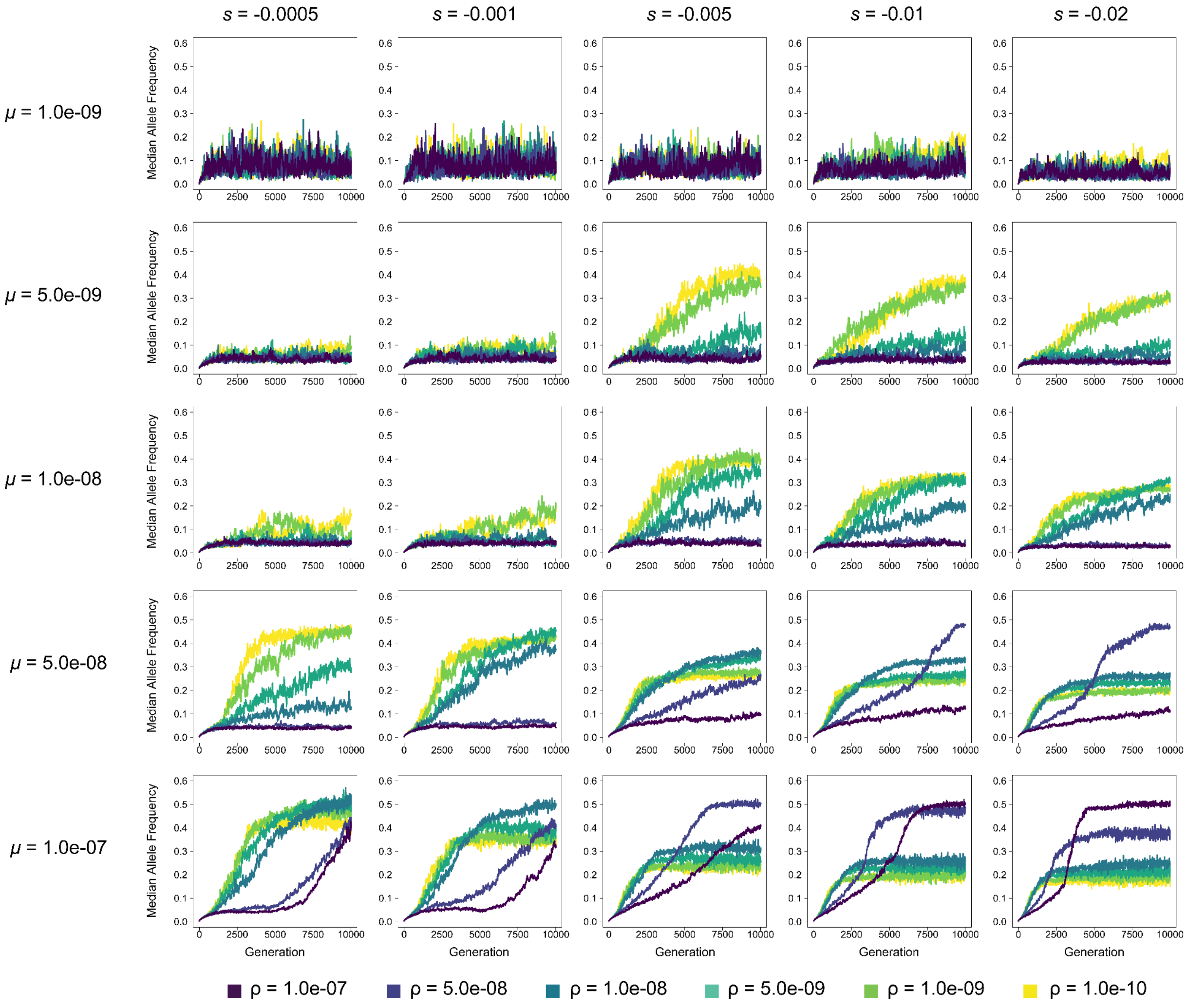

Supplemental Figure 6. Median allele frequency of diploids in each generation at varying mutation rates (μ) and selection coefficients (s) for N = 200. Each line represents the average across 10 replicates. Note: y-axis is identical across subfigures

bioRxiv preprint doi: https://doi.org/10.1101/2025.02.28.640841; this version posted March 6, 2025. The copyright holder for this preprint (which was not certified by peer review) is the author/funder, who has granted bioRxiv a license to display the preprint in perpetuity. It is made available under aCC-BY 4.0 International license.

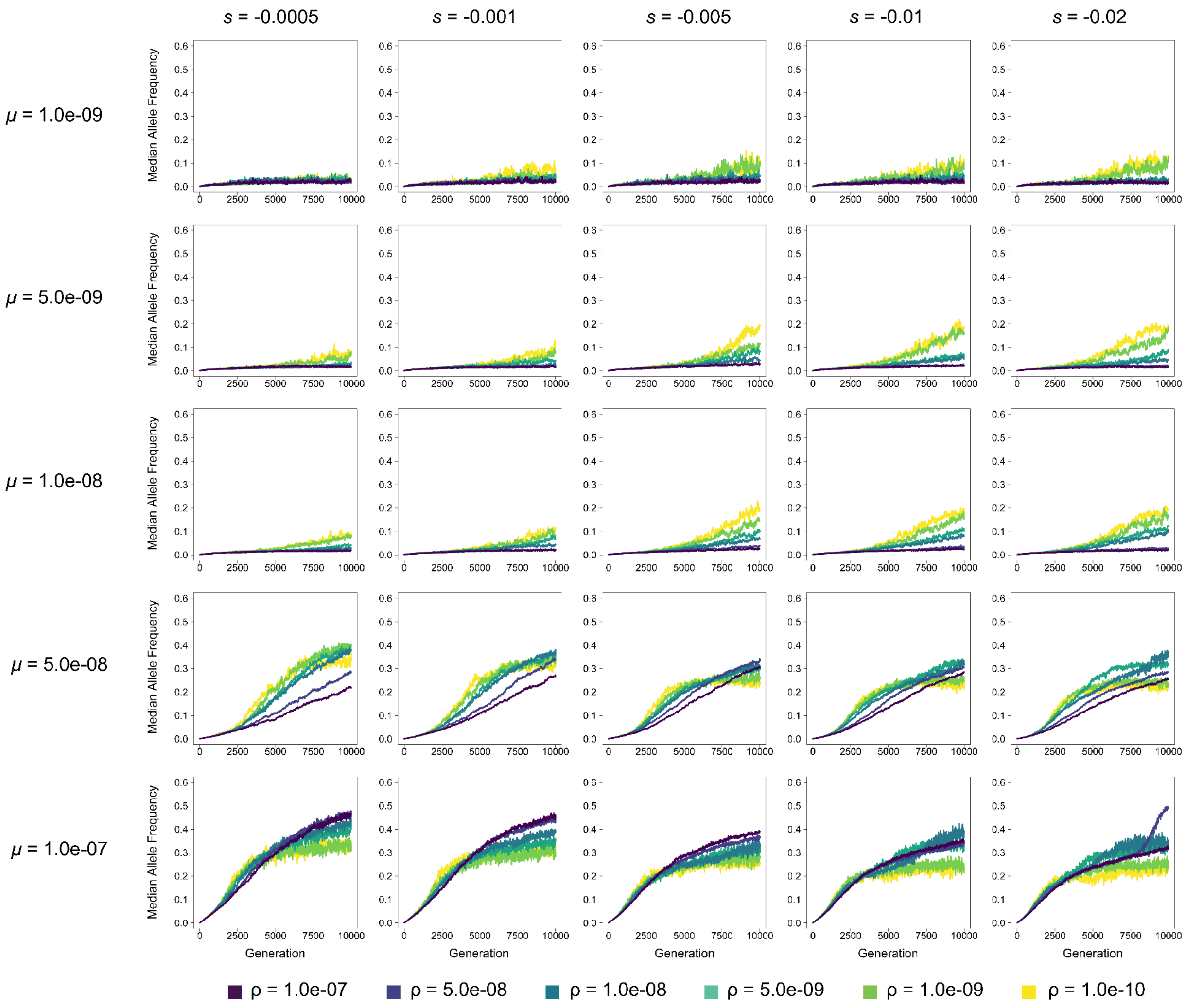

Supplemental Figure 7. Median allele frequency of autotetraploids in each generation at varying mutation rates (μ) and selection coefficients (*s*) for *N* = 500. Each line represents the average across 10 replicates. Note: y-axis is identical across subfigures

bioRxiv preprint doi: https://doi.org/10.1101/2025.02.28.640841; this version posted March 6, 2025. The copyright holder for this preprint (which was not certified by peer review) is the author/funder, who has granted bioRxiv a license to display the preprint in perpetuity. It is made available under aCC-BY 4.0 International license.

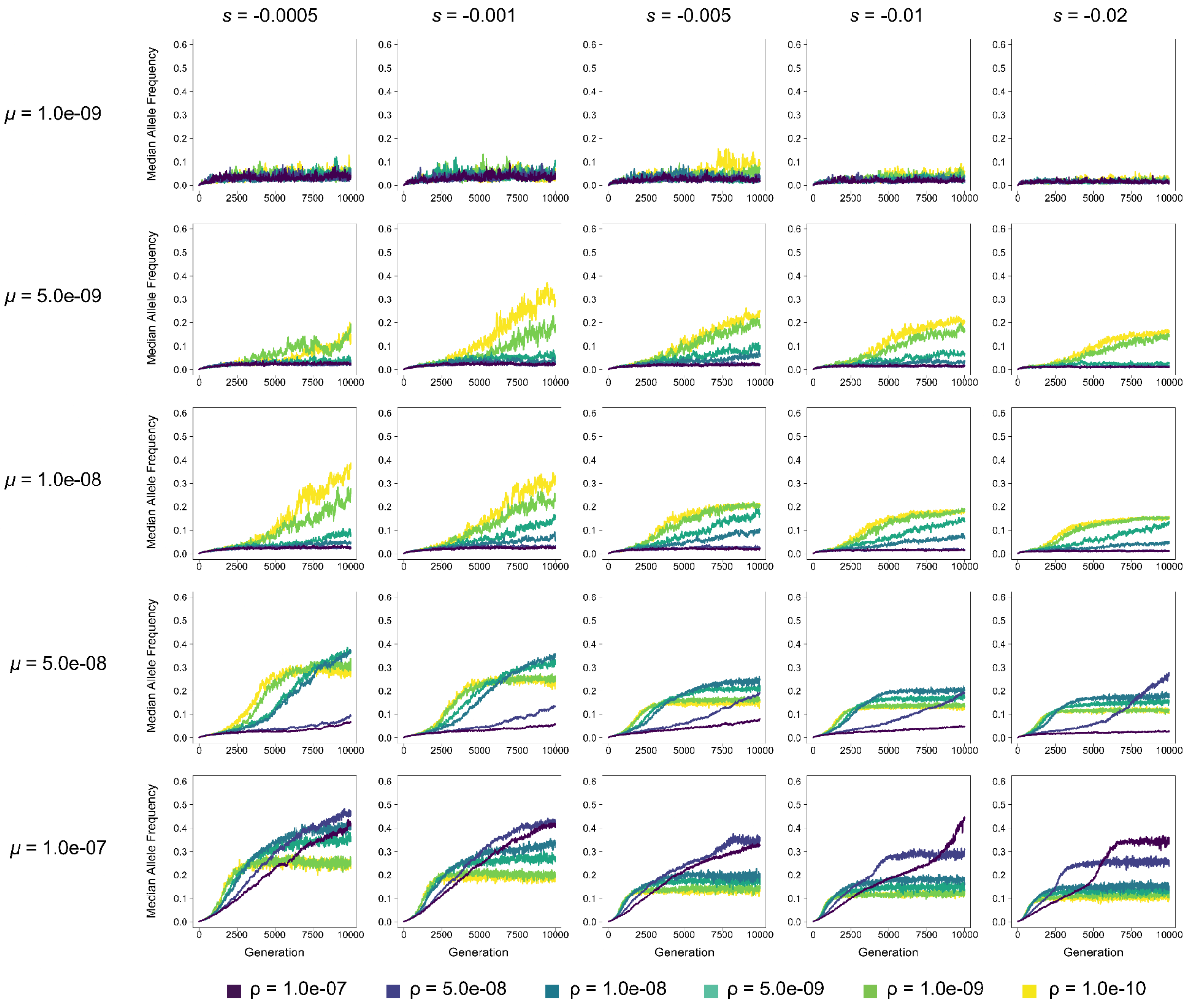

Supplemental Figure 8. Median allele frequency of diploids in each generation at varying mutation rates (μ) and selection coefficients (s) for N = 500. Each line represents the average across 10 replicates. Note: y-axis is identical across subfigures.

bioRxiv preprint doi: https://doi.org/10.1101/2025.02.28.640841; this version posted March 6, 2025. The copyright holder for this preprint (which was not certified by peer review) is the author/funder, who has granted bioRxiv a license to display the preprint in perpetuity. It is made available under aCC-BY 4.0 International license.

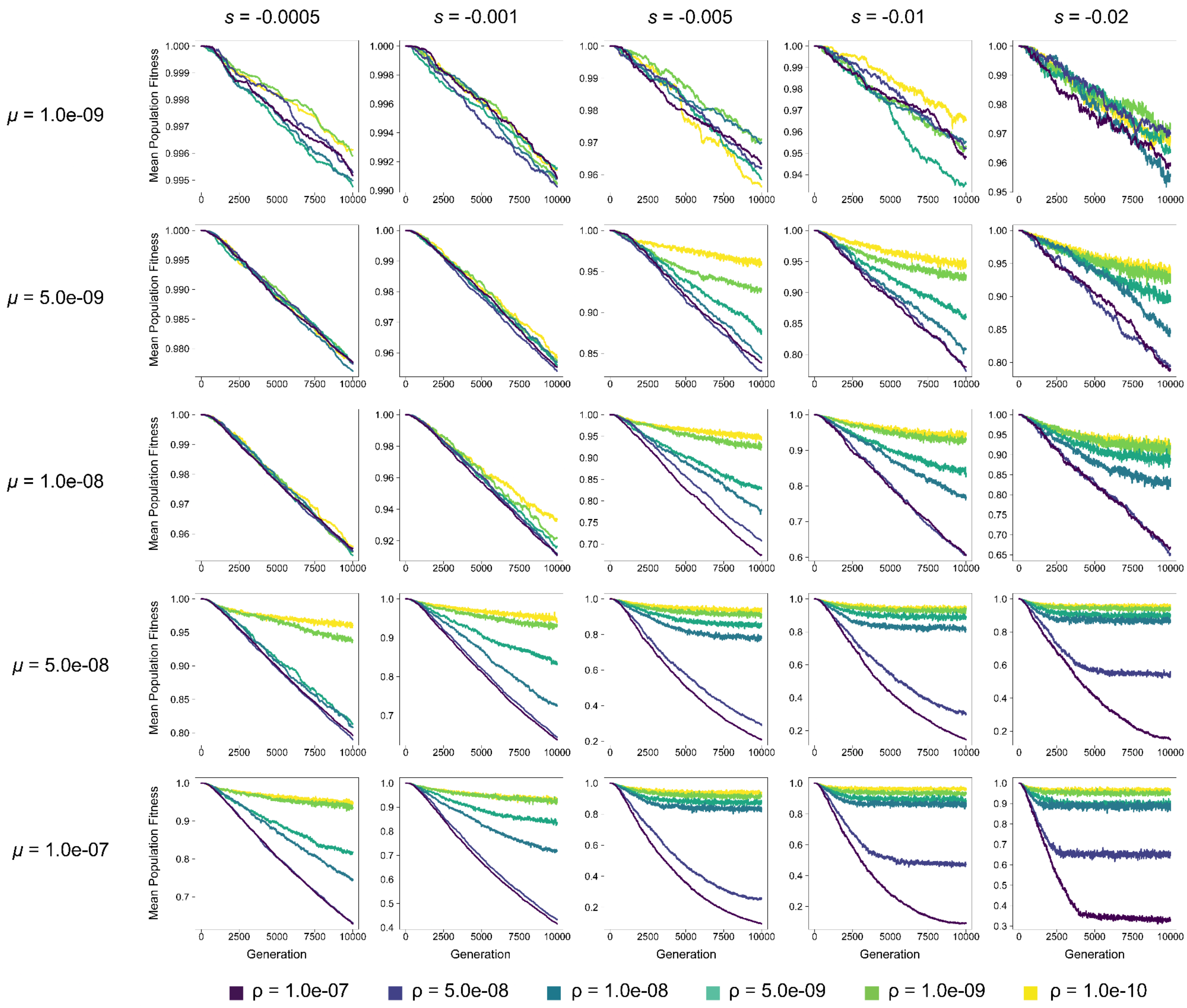

Supplemental Figure 9. Mean population fitness of autotetraploids in each generation at varying mutation rates (μ) and selection coefficients (s) for N = 100. Each line represents the average across 10 replicates. Note: y-axis varies across subfigures.

bioRxiv preprint doi: https://doi.org/10.1101/2025.02.28.640841; this version posted March 6, 2025. The copyright holder for this preprint (which was not certified by peer review) is the author/funder, who has granted bioRxiv a license to display the preprint in perpetuity. It is made available under aCC-BY 4.0 International license.

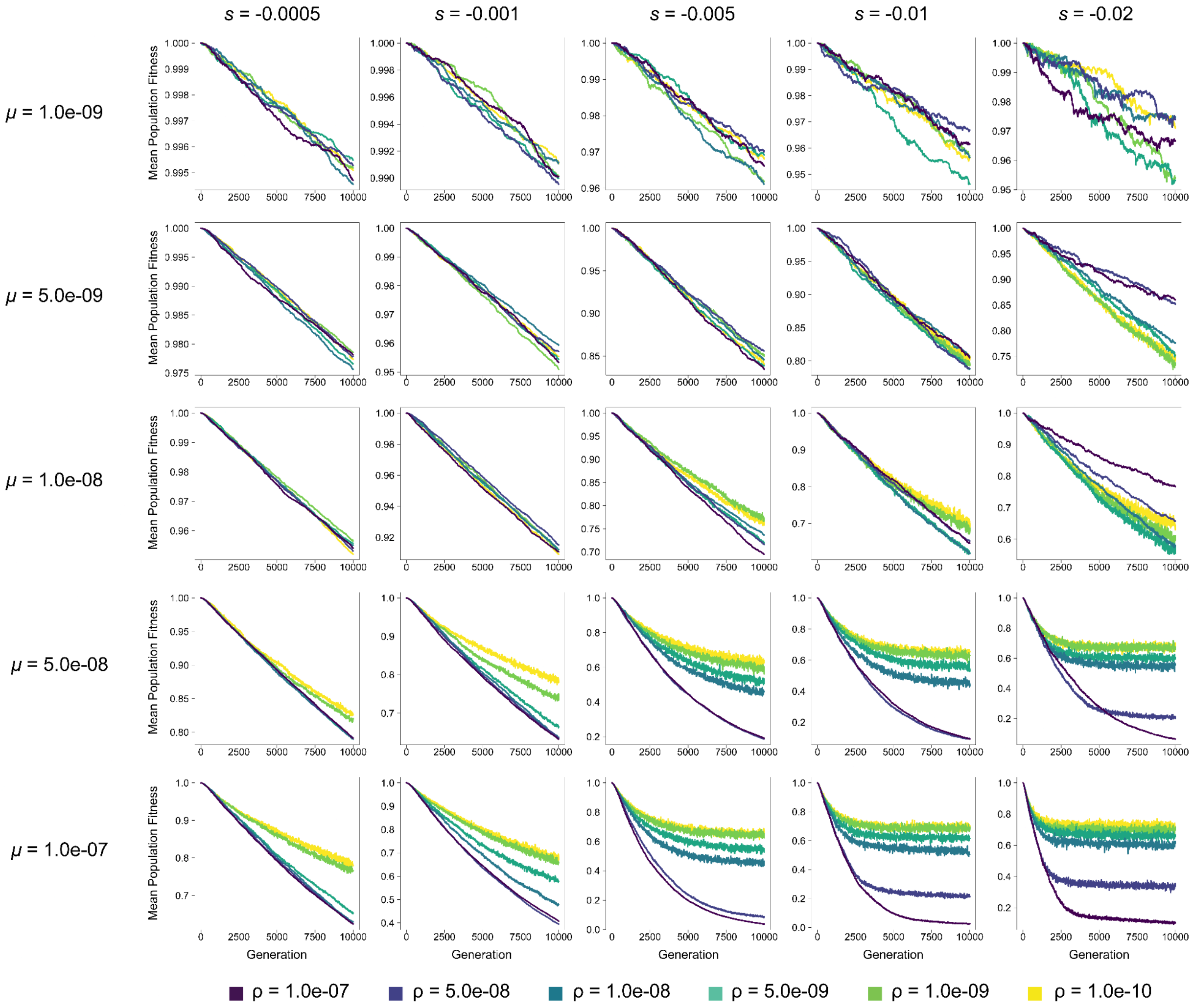

Supplemental Figure 10. Mean population fitness of diploids in each generation at varying mutation rates (μ) and selection coefficients (s) for N = 100. Each line represents the average across 10 replicates. Note: y-axis varies across subfigures.

bioRxiv preprint doi: https://doi.org/10.1101/2025.02.28.640841; this version posted March 6, 2025. The copyright holder for this preprint (which was not certified by peer review) is the author/funder, who has granted bioRxiv a license to display the preprint in perpetuity. It is made available under aCC-BY 4.0 International license.

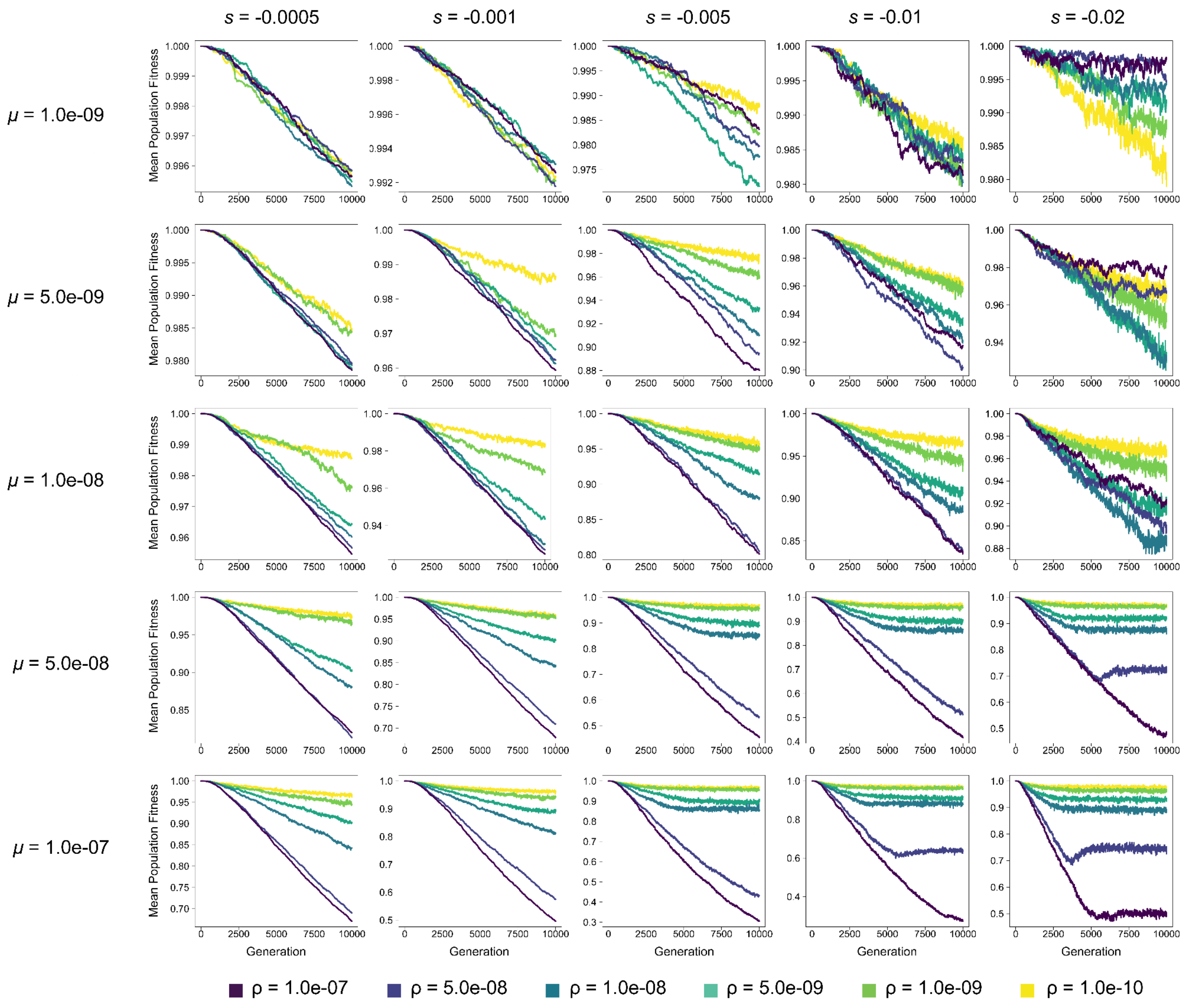

Supplemental Figure 11. Mean population fitness of autopolyploids in each generation at varying mutation rates (μ) and selection coefficients (*s*) for *N* = 200. Each line represents the average across 10 replicates. Note: y-axis varies across subfigures.

bioRxiv preprint doi: https://doi.org/10.1101/2025.02.28.640841; this version posted March 6, 2025. The copyright holder for this preprint (which was not certified by peer review) is the author/funder, who has granted bioRxiv a license to display the preprint in perpetuity. It is made available under aCC-BY 4.0 International license.

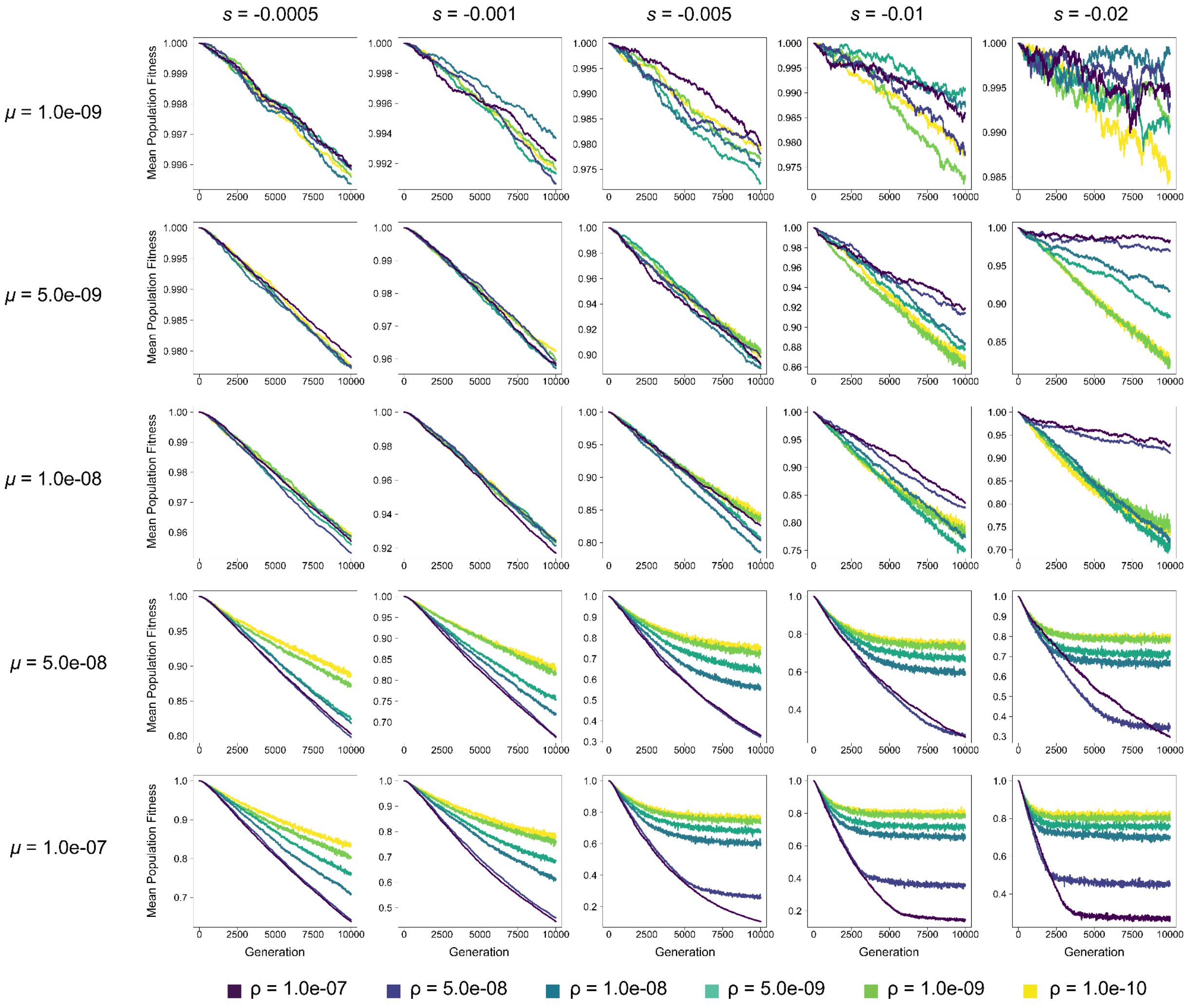

Supplemental Figure 12. Mean population fitness of diploids in each generation at varying mutation rates (μ) and selection coefficients (s) for N = 200. Each line represents the average across 10 replicates. Note: y-axis varies across subfigures.

bioRxiv preprint doi: https://doi.org/10.1101/2025.02.28.640841; this version posted March 6, 2025. The copyright holder for this preprint (which was not certified by peer review) is the author/funder, who has granted bioRxiv a license to display the preprint in perpetuity. It is made available under aCC-BY 4.0 International license.

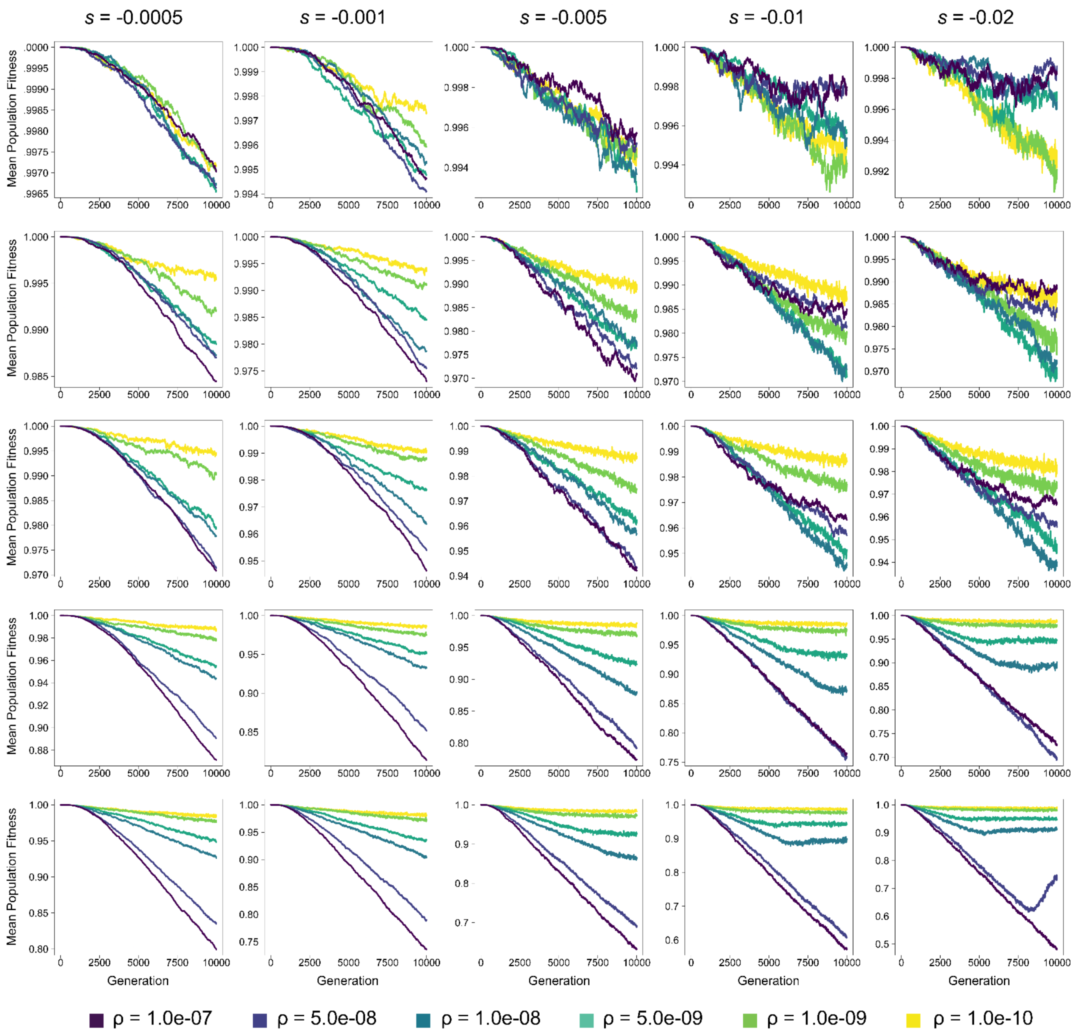

Supplemental Figure 13. Mean population fitness of autopolyploids in each generation at varying mutation rates (μ) and selection coefficients (*s*) for *N* = 500. Each line represents the average across 10 replicates. Note: y-axis varies across subfigures.

bioRxiv preprint doi: https://doi.org/10.1101/2025.02.28.640841; this version posted March 6, 2025. The copyright holder for this preprint (which was not certified by peer review) is the author/funder, who has granted bioRxiv a license to display the preprint in perpetuity. It is made available under aCC-BY 4.0 International license.

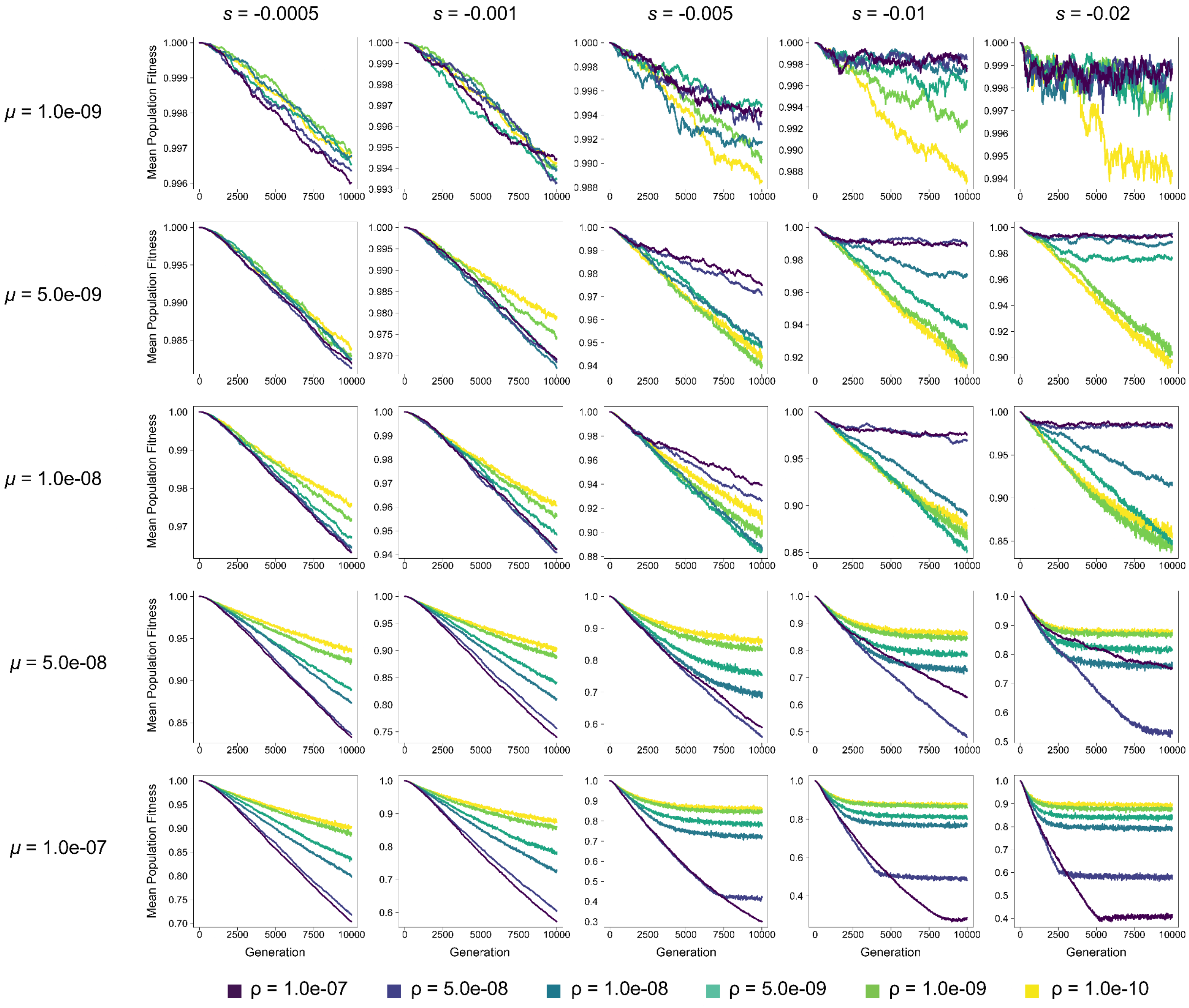

Supplemental Figure 14. Mean population fitness of diploids in each generation at varying mutation rates (μ) and selection coefficients (s) for N = 500. Each line represents the average across 10 replicates. Note: y-axis varies across subfigures.
